# A Proactive Attack Detection for Heating, Ventilation, and Air Conditioning (HVAC) System Using Explainable Extreme Gradient Boosting Model (XGBoost)

**DOI:** 10.3390/s22239235

**Published:** 2022-11-27

**Authors:** Irfan Ullah Khan, Nida Aslam, Rana AlShedayed, Dina AlFrayan, Rand AlEssa, Noura A. AlShuail, Alhawra Al Safwan

**Affiliations:** 1 SAUDI ARAMCO Cybersecurity Chair, Department of Computer Science, College of Computer Science and Information Technology, Imam Abdulrahman Bin Faisal University, P.O. Box 1982, Dammam 31441, Saudi Arabia; 2 Department of Computer Science, College of Computer Science and Information Technology, Imam Abdulrahman Bin Faisal University, P.O. Box 1982, Dammam 31441, Saudi Arabia

**Keywords:** cyber security, attacks, machine learning, Internet of Things (IoT), explainable artificial intelligence (XAI), information security, Extreme Gradient Boosting

## Abstract

The advent of Industry 4.0 has revolutionized the life enormously. There is a growing trend towards the Internet of Things (IoT), which has made life easier on the one hand and improved services on the other. However, it also has vulnerabilities due to cyber security attacks. Therefore, there is a need for intelligent and reliable security systems that can proactively analyze the data generated by these devices and detect cybersecurity attacks. This study proposed a proactive interpretable prediction model using ML and explainable artificial intelligence (XAI) to detect different types of security attacks using the log data generated by heating, ventilation, and air conditioning (HVAC) attacks. Several ML algorithms were used, such as Decision Tree (DT), Random Forest (RF), Gradient Boosting (GB), Ada Boost (AB), Light Gradient Boosting (LGBM), Extreme Gradient Boosting (XGBoost), and CatBoost (CB). Furthermore, feature selection was performed using stepwise forward feature selection (FFS) technique. To alleviate the data imbalance, SMOTE and Tomeklink were used. In addition, SMOTE achieved the best results with selected features. Empirical experiments were conducted, and the results showed that the XGBoost classifier has produced the best result with 0.9999 Area Under the Curve (AUC), 0.9998, accuracy (ACC), 0.9996 Recall, 1.000 Precision and 0.9998 F1 Score got the best result. Additionally, XAI was applied to the best performing model to add the interpretability in the black-box model. Local and global explanations were generated using LIME and SHAP. The results of the proposed study have confirmed the effectiveness of ML for predicting the cyber security attacks on IoT devices and Industry 4.0.

## 1. Introduction

With the new industrial revolution Industry 4.0, buildings are equipped with sensors, robots, and embedded systems [1]. Smart buildings play an essential role in creating a comfortable and luxurious atmosphere for people while using less energy and taking care of the environment. Despite these many advantages, the apparently growing number of Internet-connected devices (Internet of Things (IoT)) is increasingly exposed to the risks of hacking attacks. Consequently, these cyberattacks can have various harmful effects ranging from individuals, organizations or even the security system of an entire country [2]. In addition, the Internet of. Vehicles (IoV) has enabled automated driving of vehicles and green mobility. However, the performance of the models also suffers from cyberattacks. Studies have been conducted to locate the vehicle without noise and interference. Kumar et al. [3] proposed ML based algorithm for IoV security system. In addition, Shah et al. [4] has proposed an optimal routing protocol for the alerts generated for the vehicle adhoc network.

Smart building systems are easily accessible to attackers as they can be used to connect air-gapped networks to the outside world, allowing external attackers to control and transmit commands to malware installed on a target IoT network. This type of scenario is called HVACKer, which is a codename (that was given by its creators) and is able to interact with a computer’s temperature sensor in order to read temperature differences and convert them to zeros and ones (binary code). For example, ForeScout Technologies, which is a cybersecurity company, has found that thousands of insecure IoT devices in HVAC systems are vulnerable to attacks. Around 8000 connected devices, most of them in schools and hospitals, were found allowing illegal access and were highly vulnerable to attacks. HVACker’s manipulation of the HVAC systems allows them to collect private financial data and potentially store illegal data in large corporations. In places like hospitals, faulty smart HVAC systems can pose a serious threat to patients who need to recover at certain temperatures and airflows [2]. In addition, with the advent and advancement ML algorithms, several studies have been conducted, aimed at developing a method that can detect and classify different types of cyber-attacks in general and also targeted at HVAC systems [5].

Most previous studies on HVAC attack detection have achieved the best results using the deep learning model. The main limitation of the DL model is that it requires huge amount of data to train the model and is computationally intensive. In addition to this the nature of the DL models is black box. The internal reasoning of the DL models is hidden. Similarly, some of the ML models such as Support Vector Machine (SVM), Artificial Neural Network (ANN), and Ensemble models like Random Forest (RF), Gradient Boosting (GB), Ada Boost (AB), etc. also suffered from black box nature. However, the advent of of explainable artificial intelligence (XAI) has alleviated this disadvantage by providing a post-hoc approach to add the explainability to the back box nature models. Recently, XAI has been used in various real-world applications such as healthcare, business, engineering and cybersecurity etc. [6,7]. However, to the authors knowledge, XAI has not been used in the HVAC system attack detection. Therefore, in the current study, the post-hoc XAI approach was used to make the proposed model transparent.

Previous studies focused solely on the security breaches of the BMS network as the detection target and not the HVAC system itself, some of which had used IoT-based security systems and access control strategies in the building devices, authorization, and authentication systems to protect the service access. In contrast, others used a multivariate correlation analysis technique that examines each packet traversing the network for the attacks stored in the database. Nowadays, a large number of studies have been conducted solely focused on how to prevent HVAC cyberattacks, one of these studies was conducted by Elnour et al. [8] using the Isolation Forest (IF) technique which is an unsupervised ML algorithm used in the data-driven attack detection strategy along with Convolutional Neural Network (CNN) to encode the feature extraction of temporal data. Isolation Forest works to detect abnormal activity by distinguishing between normal and abnormal. Their model had successfully detected many critical attacks, resulting in a 0.61 recall and 0.90 precision. Furthermore, the main goal of the proposed study is to develop an effective and interpretable detection model that could detect attacks on HVAC systems.

The main contribution of the proposed study is articulated as follows:

- Proposing a proactive model with improved performance to detect attacks on HVAC systems.
- Conduct a comprehensive comparison of multiple ML models such as Decision Tree (DT), Random Forest (RF), Gradient Boosting (GB), Ada Boost (AB), Light Gradient Boosting (LGBM), Extreme Gradient Boosting (XGBoost), and CatBoost (CB). The best results were obtained with XGBoost.
- To the best of author’s knowledge, the proposed study is the first study to have used explainable artificial intelligence (XAI) to detect attacks on HAVC systems.
- The proposed model achieved the best result compared to the benchmark study with the reduced features and better explainability.
- To alleviate the data imbalance SMOTE and TomekLink were used.

The organization of the research is as follows. Section 2 contains previous studies related to the proposed study. Section 3 presents the material and methods used in the current study. Furthermore, the experimental setup and the results are presented in the Section 4. While Section 5 contains further discussions and finally Section 6 contains the conclusion of the proposed study.

## 2. Review of Literature

Recently, due to the digital transformation of air conditioning systems, HVAC systems have recently become more vulnerable to many cyber-attacks. As a result, some studies have been made on the safety measure for HVAC systems. Wu et al. [9] focused on hidden voice attacks and how such security breaches can affect many organizations and users. They proposed a fusion-based method to combine normal and obfuscated samples, since a hybrid hidden voice attacks commands and effectively fools defensive classifiers. In addition, they changed the sample speed, pitch, and time scale distortion to make them more confusing for human while ensuring machines can still recognize them. Furthermore, experiments showed that hidden voice attacks are robust and generalizable in various realistic scenarios. The results showed that the proposed hidden voice attack commands can successfully bypass ML-based security techniques in various real-world scenarios with an average success rate of 0.941.

In addition, a study by Munir et al. [10] used an unsupervised anomaly detection technique using time series data acquired by the authors over 2.5 years. The proposed method uses a knowledge base that incrementally stores the HVAC system’s normalized time-series data points. The proposed strategy has the advantage of detecting long-term and short-term anomalies that traditional approaches failed to detect. Furthermore, the algorithm was compared to several state-of-the-art algorithms and outperformed with precision of 0.91 and a recall of 0.80.

Also, Novikova and Bestuzhev [11] proposed a solution for HVAC data that uses image similarity analysis to automate the search for days with similar HVAC working patterns. The main idea was to create a graphical representation of the daily HVAC data and then compare it to the Structural Similarity Index (SSIM) for each image produced to examine the similarity. Days with a high level of dissimilarity are considered abnormal and require immediate attention. The proposed method has been tested tested on VAST MiniChallenge-2 2016 dataset, including HVAC logs. Their experiments showed that days with abnormal function had three times higher measure of dissimilarity (DSSIM) than days with normal function. In addition, the accuracy of their developed approach is highly dependent on data availability.

Ashraf et al. [12], introduced IoTBoT-IDS, a new botnet detection system for IoT-based networks that uses statistical learning-based approaches, such as the Beta Mixture Model (BMM) and a Correntropy model, to predict the normal behavious for IoT capture networks. Any deviation from normal behavior is defined as abnormal. Furthermore, a statistical model was used for the feature extraction to represent network data while improving botnet classification. In addition, three benchmark datasets from real IoT networks were used to test IoTBoT-IDS. They found that the proposed detection system had achieved an accuracy of 0.992, outperforming other traditional intrusion detection approaches by 2–5%.

Similarly, Chakraborty et al. [13] performed a comparative analysis of different functional and non-functional methods to extract the features in order to accurately tracking real-time industrial IoT attacks. To investigate the behavior of an IoT system, a lab-controlled set of cyberattacks on a Secure Water Treatment (SWaT) system was used. They proposed a detection approach based on functional shape analysis (FSA), that uses the data to extract features that can capture the waveform profile and improve the classifier’s performance. When projecting IoT threats, their technique demonstrates a trade-off between functional and non-functional strategies.

Furthermore, Liu et al. [14] examined some attacks that could affect sensor nodes and networks in IoT scenarios using the NSL-KDD dataset. they also examine eleven ML techniques and provide the results to identify the attacks introduced. They found that ensemble methods and tree-based approaches outperformed the other ML methods. They found that XGBoost had the highest score with an accuracy rate of 0.97.

In addition, Vargas et al. [15] proposed an integrated protection mechanism for networks of IoT devices using the UNSWNB15 dataset by combining blockchain algorithms and ML techniques. The model was proposed for threat detection and activation of secure information procedures. For the ML, K-Nearest Neighbor (KNN) was used for the classification. Several types of attacks such as fuzzers, DoS, backdoor etc. were covered in their study, with the best results for fuzzers attack with an accuracy rate of 0.99.

Identifying their key characteristics is crucial to detecting malicious attacks. Therefore, Ahsan et al. [16] developed a feature selection algorithm for filtering insignificant variables called Dynamic Feature Selector (DFS). Feature selection algorithms such as person correlation, XGB feature importance and information gain were used. The study used two datasets NSLKDD and UNSW-NB15. Several classifiers were compared and combined such as Bi-LSTM, RF, CNN-LSTM, Gated Recurrent Units (GNU) and DT. The bagging method was used to combine the base learner’s decision. RF outperformed the other classifiers for NSLKDD and UNSW-NB15 datasets with an accuracy of 0.9965 (NSLKDD) and 0.9276 (UNSW-NB15). For NSLKDD dataset, the feature set was reduced to 50%. Many studies have recognized how destructive False Data Injection Attacks (FDIA) are for AC systems and create defensive mechanisms. One of them was developed by Dehghani et al. [17] to build a new mechanism that detects any erroneous data injection in the AC-state estimation (SE) based on a signal processing technique. The proposed method is based on the analysis of successive states of the system by single wavelet entropy (SWE). The approach is used in different case studies to identify cyberattacks with different types of false data injection. The simulation results showed that the proposed FDIA detection method perform outstanding and achieving an accuracy rate of 0.965.

Another study was conducted to prevent FDIA of AC systems reported by Yu et al. [18] and propose a novel FDIA detection technique aimed at assessing the AC state. When malicious data is introduced into the state vectors, the temporal and spatial data correlations may differ from those found under normal operational settings. The proposed technique used wavelet transform and deep neural network methods for detection. Furthermore, the performance of the proposed approach was evaluated using extensive case studies of 300-bus and IEEE 118-bus power systems. The results outperformed conventional FDIA detectors and showed that the developed mechanism can detect attacks with greater than 0.90 accuracy.

Furthermore, Ding et al. [19] developed a DL-based classification scheme to detect and classify FDIA using the dataset consisting of 30,000 measurements. The proposed model used a conditional deep belief network (CDBN) integrating both conditional gaussian-bernoulli RBM (CGBRBM) with the conventional DB. Furthermore, two realistic adversary attacks were considered to test their model: the least-effort and multiple attacks. The study achieved an accuracy of 0.984 in verifying multiple scenarios and more than 90% for robustness and scalability verification. Ultimately, the proposed model surpassed the accuracy and robustness of both the Support Vector Machine (SVM) and Multilayer Perceptron (MLP) for detecting FDIA.

Campi et al. [20] introduced a speech biometric framework for cyber-attack mitigation f to distinguish between synthetic and authentic human voices. They used the empirical mode decomposition (EMD) method in combination with the mel-frequency cepstral coefficient (MFCC) and SVM classifier. They used three different datasets for their experiments with, 960, 1152, and 12,624 sentences. Furthermore, their method outperformed other methods with an accuracy of over 0.90, which is crucial for biometric security.

Inaddtion, Elnour et al. [8], presents a HVAC system model consisting of 12 zones created with the Transient System Simulation Tool (TRNSYS), which was utilized to analyze the cybersecurity of HVAC systems. The dataset wis divided into two logs containing regular operational data logs with a total of 194,301 samples in the first log and 8840 in the second log. The data was collected and used to build and test a semi-supervised data-driven attack detection technique namely Isolation Forest (IF). The proposed technique is compared to traditional ML algorithms and showed promising results in attack detection with high reliability and minimal computational cost, achieving a precision of 0.90 and a recall of around 0.61.

In summary, the studies to date have used different datasets and methods to detect attacks and anomalous traces and visualized their results. However, few studies have applied ML algorithms and there is a need for further improvements and investigations. Table 1 contains the summary of the previous related studies. Finally, in our study, we aim to propose an interpretable model for the proactive detection of attacks specifically related to HVAC systems. Therefore, in the current study we will use the HVAC dataset published by Elnour et al. [8]. Based on the author’s knowledge there is only one study that used the current dataset. One of the limitations of the research [8] was that the proposed solution could only identify attacks that adversely affect system function by changing the temperature comfort levels and/or generating extreme energy consumption. Also, the study only represented the performance of its proposed algorithms using only used two evaluation measure namely, precision and recall. In the current study, we will apply and evaluate several ML algorithms. Furthermore, we aimed to propose a model with the enhanced performance compared to the benchmark study. We attempt to develop an accurate and interpretable model using ML and XAI.

## 3. Material and Methods

**Figure 1:**
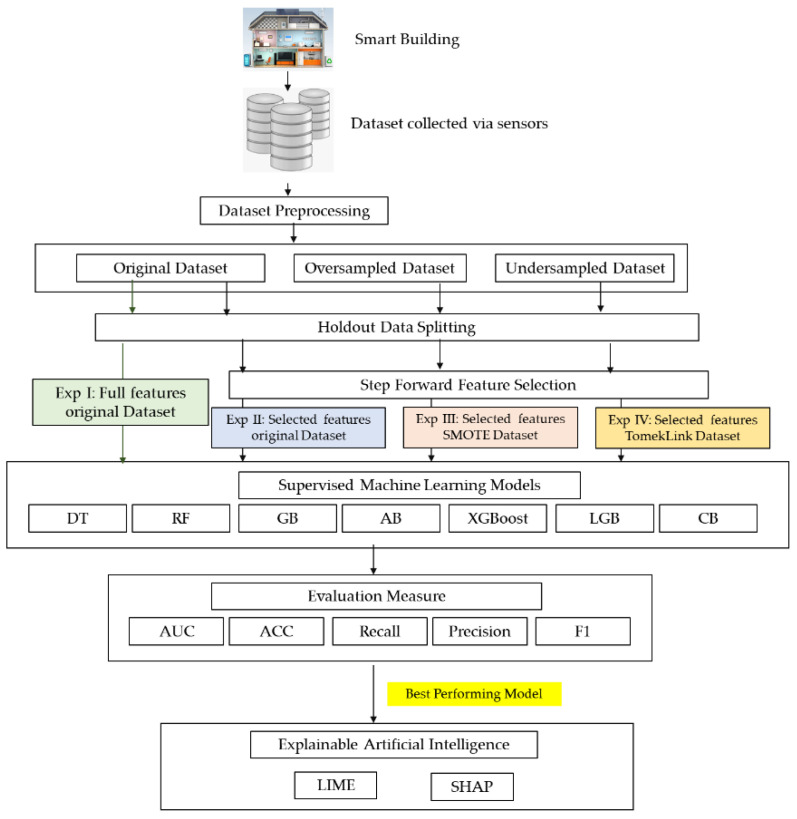
Proposed methodology framework.

The growing number of cyber-attacks has created a need for automated and effective methods for the detection of cyber security attack detection methods. The attacks in the smart BMS are related to the increase in the energy consumption or the abnormal increase or decrease in the temperature. Such types of attacks affect the comfort and the performance of the smart building. The aim of this study is to propose a proactive ML based detection model for the attacks related to smart HVAC systems, which are considered as a crucial element of the BMS. This section of the study contains the methodology used in the current study i.e., the dataset description, preprocessing, feature selection, description of the classifiers used, and evaluation measures. Figure 1 contains the framework of the proposed methodology. Firstly, the dataset collected via sensors was pre-processed, after preprocessing due to the data imbalance by both oversampling using SMOTE (Synthetic Minority Oversampling Technique) and TomekLink undersampling was performed. The classification models were trained and tested with original data without any sampling technique, oversampling and undersampling dataset. Each dataset is divided into training and testing using the holdout data splitting method. Furthermore, to identify significant features, a step-forward feature selection technique was performed. The study also examined the performance of the algorithm with and without the feature selection. Seven ML algorithm such as Decision Tree (DT), Random Forest (RF), Gradient Boosting (GB), Ada Boost (AB), Extreme Gradient Boosting (XGB), Light Gradient Boosting (LGB) and Cat Boost (CB) were used. To compare and evaluate the performance of the proposed algorithms several evaluation measures such as accuracy (ACC), precision, recall, F1-score and Area Under the Curve (AUC) were used. Explainable Artificial Intelligence (XAI) was performed on the best performing model using Local Interpretable Model Agnostic Explanation (LIME) and SHapley Additive explanation (SHAP).

### 3.1. Dataset Description

The study was performed using the Elnour et al. [8] HVAC systems dataset. The dataset was acquired using the Transient System Simulation Tool (TRNSYS). This tool presents the behavioral simulation of dynamic systems using mass balance, energy, and mass balance equations. The data covers a three-floor, 12-zone cooling system and includes 3 logs: dataset log 1 is a four-month log of normal operational data, whereas dataset log 2 is a 20-day log of data. Finally, log 3 contains data holding both regular and malicious, injected through 20 days. Table 2 contains the details of the 3 log files i.e., number of samples and features, and type of attack. In all the three logs contains similar variables that could aid the attack detection process are included (as shown in Table 3), such as hour of year and day, sensor’s temperature measurements, control signals, temperature setpoint, comfort indices, total estimated power consumption by the HVAC system and finally the system status (i.e., 0 for normal operation and 1 for attack). The system status, the targeted class, is the only binary attribute, while all other classes are numerical. Table 2 and Table 3 below show information about the dataset logs and its features.

Furthermore, data log 3 contains 16 different injected attacks, which differ in the zone or floor in which they occur, but can be classified primarily into four types:

1. Changing the control system’s setpoints.
2. Freezing sensor readings or adding a bias, which falsifying sensor readings.
3. Control signals are falsified by freezing their values or creating a bias.
4. Modifying component command signals.

In the current study, a combination of both log 2 and log 3 is used, and since some of the attributes have redundant names, they have been renamed to avoid duplicating in terms of feature name.

### 3.2. Feature Selection

Feature selection is a step that refers to the process of minimizing the number of input variables that can be used to develop the predictive model with the best results. The number of input variables should be reduced in order to decrease the computational cost of modeling and in some cases, to increase the model’s performance [21]. There are several techniques used for feature selection which are all designed to improve the learning process of the model. However, this study used a step forward feature selection technique to select only the significant features needed to train the model. Step forward feature selection is a technique that begins by evaluating each feature and selecting the one that yields the highest performance of the model. A five-fold cross-validation was applied to assign the best features for the models to be used. Initially, the classifiers had a higher performance rate as the number of features increased. The selected features were as follows, ‘B_Tz2’, ‘B_Tz4’, ‘C_Tz1’, ‘C_Tz3’, ‘C_Tz4’, ‘T_t’, ‘B_Uz4’, ‘C_Uz4’, ‘AA_Tz1’, ‘AA_Tz2’, ‘AA_Tz3’, ‘AA_Tz4’, ‘BB_Tz1’, ‘BB_Tz2’, ‘BB_Tz3’, ‘BB_Tz4’, ‘CC_Tz1’, ‘CC_Tz2’, ‘CC_Tz3’, ‘CC_Tz4’, ‘Tt’, ‘T_chiller’, ‘BBB_z3’, and ‘CCC_z4’. Table 4 shows list of selected features. The value of the parameters for the step forward feature selection were k-features = 24, forward = true, floating = false, verbose = 2, scoring = accuracy, cv = 5 and n_jobs = 6.

### 3.3. Classification

#### 3.3.1. Decision Tree

Decision trees (DT) is a non-parametric supervised learning approach method for regression and classification. This method aims to learn basic decision rules from data attributes in order to develop a model that predicts the value of a specific target variable. The decision criteria become more complicated as the tree grows deeper, and the model becomes more accurate [21].

The DT algorithm builds a tree by applying various criteria to its branches. It consists of a root node (the starting point), internal nodes (where the split occurs), and leave nodes (also known as terminal or final nodes).

The selection of the attributes in a decision tree must be done with two important measures: namely, entropy and information gain. Entropy measures the degree of uncertainty for a random variable, while information gain is basically the measure of change in entropy after the data has been partitioned on a specific attribute. The attribute with the highest information gain is placed at the top of the decision tree. The following equation below shows how entropy is calculated, where *E(S)* represents entropy:

(1) $$E\left(S\right)=\sum_{x\epsilon N}^{\;}p\left(x_{\;}\right)\log_{2}p\left(x_{\;}\right)$$

where $p\left(x_{\;}\right)$ denotes the probability of an attribute x, and *N* is the complete set of attributes in the dataset together with the target class. The following equation shows how the information gain is calculated, where *I*(*S*,*A*) represents the information gain:

(2) $$IG\left(S,\;A\right)=E\left(S\right)-\sum_{i=0}^{n}p\left(x_{i}\right)\times E(S,N)$$

where *E*(*S*, *N*) represents the entropy of the attribute with the target class, and *E*(*S*) denotes the entropy of the target attribute.

#### 3.3.2. Random Forest

The Random Forest (RF) algorithm and the DT algorithm are similar. RF consists of many individual DTs. RF is an ensemble learning method and is known for one of the most widely used algorithms for solving multi-classification and prediction tasks. It combines random subspace and bagging ensemble learning. Bagging is a bootstrap aggregation method that uses a simple random sample with replacement for imbalanced datasets. In bagging, and as RF’s name suggests, it follows a random mechanism when generating results. Unlike decision trees, RF provides a fixed-size subset of all the attributes; a tree of all properties is not generated at all times. This significantly reduced the computational cost [22]. The conditional characteristics of a RF are determined individually by each tree. When a sample reaches a root node, it is propagated to all sub-trees. Each sub-tree anticipates the class label for that specific sample. Finally, the highest class is assigned to that sample. The main procedure of RF includes the following steps

Step 1: The sample is first randomly selected from a dataset using the simple random sampling bootstrapping resampling approach.

Step 2: A selection of attributes from all the attributes in the dataset will be generates a random subspace.

Step 3: Using bootstrap sampling, a tree is constructed using the ID3 algorithm and performs a voting mechanism on each predicted result. 

Step 4: Repeat steps 1–3 until it generates a sufficient number of trees to reach the desired result, and then select the prediction with the highest votes as the final prediction.

Suppose *S* is a dataset with $X=\left\{x_{1},\;x_{2},\;\ldots ,\;x_{n}\right\}\;$features and a target class label *y*. This method is a standard representation of the random forest. When training a group of classifiers, RF is expressed by the following equation.

(3) $$D={\left\{\left(x_{i},y_{i},\right)\right\}}^{n}i=1$$

The Gini index is used to assess the quality of the split. The node’s *GI* is determined by

(4) $$GI\left(N\right)=\sum_{i\neq j}P\left(\omega_{i}\right)P\left(\omega_{j}\right)$$

where *P*(*i*) indicates the ratio of entities in the dataset for a class category *i*.

#### 3.3.3. Gradient Boosting

Gradient Boosting (GB) is an ensemble based supervised machine learning algorithm that is used for classification and regression. It is an ensemble method that uses many weak learners to construct a powerful regression and classification model. GB is based on the assumption that combining the best possible next model with the previous models reduces the overall prediction errors [23].

GB comprises of three basic components: first, the loss function is evaluated to understand how good the model is making predictions based on data available to the model. Secondly, the weak learner tries to classify data but with poor performance and high error rate. Thirdly, an additive model is used to modify the model in a sequential and iterative method by adding previous weak learners one by one, improving the model while reducing the bias error in each cycle. The flexible algorithm has demonstrated good prediction accuracy and is excellent for dealing with missing data. Despite all of its benefits, GB is computationally intensive and continues to be improved to avoid errors that could overemphasize outliers and cause overfitting [24].

#### 3.3.4. Ada Boost

AdaBoost (Adaptive Boosting) (AB) is an ensemble-based ML algorithm used for classification. However, it is best used to improve DT performance on binary classification tasks. These trees are often referred to as decision stumps [25]. This algorithm builds a model and assigns equal weights to all data points. It then applies larger weights to misclassified points. Next, all the points with larger weights are given more relevance in the next model. It will continue to train models until a lower error is returned. Ada boost’s main drawback is that it requires a high quality dataset, which means that before using an Adaboost algorithm, the dataset must be free from any noisy data.

#### 3.3.5. Light Gradient Boosting

The light gradient boosting model (LGBM) is a gradient boosting framework that uses a tree-based learning method. The distinguishing feature of the algorithm from other tree-based algorithms is that the trees grow vertically while other algorithms grow horizontally. This implies that LGBM grows the tree leaf-wise and selects the leaf with the smallest delta loss, whereas other algorithms grow them levels-wise. In addition, leaf-wise algorithms can minimize loss more than level-wise algorithms while expanding the same leaf. LGBM is referred to as ‘Light’ due to its reduced speed, ability to process massive amounts of data, and minimal memory requirements. However, as more features are included in the data, efficiency and scalability remain inadequate. Furthermore, since LGBM splits the tree leaf-wise, which means creating more complicated trees, it can sometimes lead to overfitting [24].

#### 3.3.6. Extreme Gradient Boosting

Extreme Gradient Boosting (XGBoost) is a scalable ensemble-based ML algorithm developed by University of Washington research project. It is used for structured or tabular data and to solve various ML problems quickly and correctly [26]. XGBoost built the decision trees sequentially, with significant weights. All the independent variables are given weights, which are then fed into the DT that will predicts the outcomes. If the weight of variables that are misclassified by the tree is increased, those variables are fed into the second DT. After that, the individual classifiers are integrated to create a more powerful model. Ranking, regression, custom prediction, and classification are some of the well-known uses of XGBoost.

#### 3.3.7. CatBoost

CatBoost (CB) is derived from two words, “Categorical” and “Boosting”. It is an open source ML algorithm developed by Yandex [27]. It can be used with a variety of data formats to help organizations solve common challenges and achieve best-in-class accuracy. Specifically, it is powerful in two distinct ways: it delivers cutting-edge insights without the extensive data training that other ML algorithms require, and it offers robust support for descriptive data formats that often accompany business problems.

The algorithm can process a variety of data formats, including images, audio, video, and historical information. The name “Boost” derives from the gradient boosting ML algorithm, as this algorithm is based on it. Gradient boosting is a sophisticated ML approach that has been successfully applied to a variety of commercial difficulties such as fraud detection, item recommendations, and forecasting.

Table 5 shows the list of parameters for the proposed classifiers after optimization

### 3.4. Evaluation Measures

To compare the performance of classifiers, several evaluation measures such as Area Under Curve (AUC), accuracy (ACC), precision, recall, and F1-score were used. The evaluation metrics formula is shown in the equations below.

$$ACC=\frac{TP+TN}{TP+FN+TN+FP}$$

$$Precision=\frac{TP}{TP+FP}$$

$$Recall=\frac{TP}{TP+FN}$$

$$F_{1}=\frac{2\times precision\times recall}{precision+recall}$$

where true positive (*TP*) indicates the sample that is attack and also predicted as attack by the proposed model. True negative (*TN*) indicates the non-attack labeled sample predicted as non-attack. False positive (*FP*) indicates the sample that is originally non-attack but predicted as attack by the model. While false negative (*FN*) indicates the sample that is originally attack but predicted as non-attack by the model. *ACC* represents the ratio of the number of correctly predicted samples to the total number of samples used during the testing phase. Precision indicates the usefulness of the proposed model, while recall indicates the completeness of the proposed model. While F1 indicates the ratio of precision and recall of the model. The AUC evaluation measure indicates the quality of the proposed model, a higher the value indicates better performance.

## 4. Experiments and Results

The models were implemented using the Python environment (ver. 3.9.7) and several libraries were used such as Sklearn library to implement the classification models. Additional libraries were used, such as NumPy (ver. 1.19.5) for processing numeric and array data, Pandas (ver. 1.3.4), it is a data frame and is used for data management, and Matplotlib (ver. 3.4.3) for data visualization. While XAI was implemented using SHAP (ver. 0.41.0) for global explanation and LIME (ver. 0.2.0.1) for local explanation. The dataset used in this study was split into training and testing by the hold out method in a ratio of 75:25. We utilized several classification models such as DT, RF, GB, AB, XGBoost, LGBM, and CB to detect attacks on HVAC systems based on log information. However, for the feature selection step forward feature selection algorithm was used. AUC, ACC, precision, recall, and F1 scores was used to evaluate the proposed model’s performance. Furthermore, grid search method was used for model optimization. Several experiments were performed to investigate the effects of feature selection and data sampling using SMOTE oversampling and TomekLink undersampling. The following list of experiments was performed.

- Experiment I: Training and testing the classifiers using all the features in the original dataset
- Experiment II: Training and testing the classifiers using the selected features and the original dataset
- Experiment III: Training and testing the classifiers using the selected features and the SMOTE oversampled dataset
- Experiment IV: Training and testing the classifiers using the selected features and the TomekLink undersampled dataset

In general, the XGBoost classifier performed best compared to the other classifiers. In addition, most of the proposed classifiers have achieved results that have outperformed the benchmark study. Table 6 shows the test result of the classifiers using the complete dataset. While Table 7 contains the results of experiment II.

### 4.1. Handling Data Imbalance

In the domain of ML, classification problems are extensively investigated. The input data or predictor is assumed to predict the class label in the classification task, when the target or output is a categorical variable. One of the most common classification problems is to have an imbalanced class dataset where the number of observations for one of the target class labels differs significantly from other class labels. The current HVAC attack detection dataset suffers from imbalance. There are two class labels in the dataset, namely the class ‘0’, which refers to ‘not attack’, and is extremely smaller than class ‘1’, which refers to ‘attack’, since the difference between them exceeds 30,000.

The dataset used in the current study has an extrinsic imbalance type that occurs when a dataset or data analysis is limited by storage, time, or other factors. To deal with the imbalanced dataset, various resampling techniques was experimented such as SMOTE oversampling and TomekLink undersampling. In oversampling, the synthetic data for the minority class is generated such that the number of samples in the minority class will be equal to the number of samples in majority class. However, undersampling reduces the number of samples for the majority class is reduced to balance the dataset. Table 8 contains the testing result of the proposed classifiers using SMOTE oversampling with selected features. Table 9 shows the test results of the proposed models with TomekLink undersampling.

**Figure 2:**
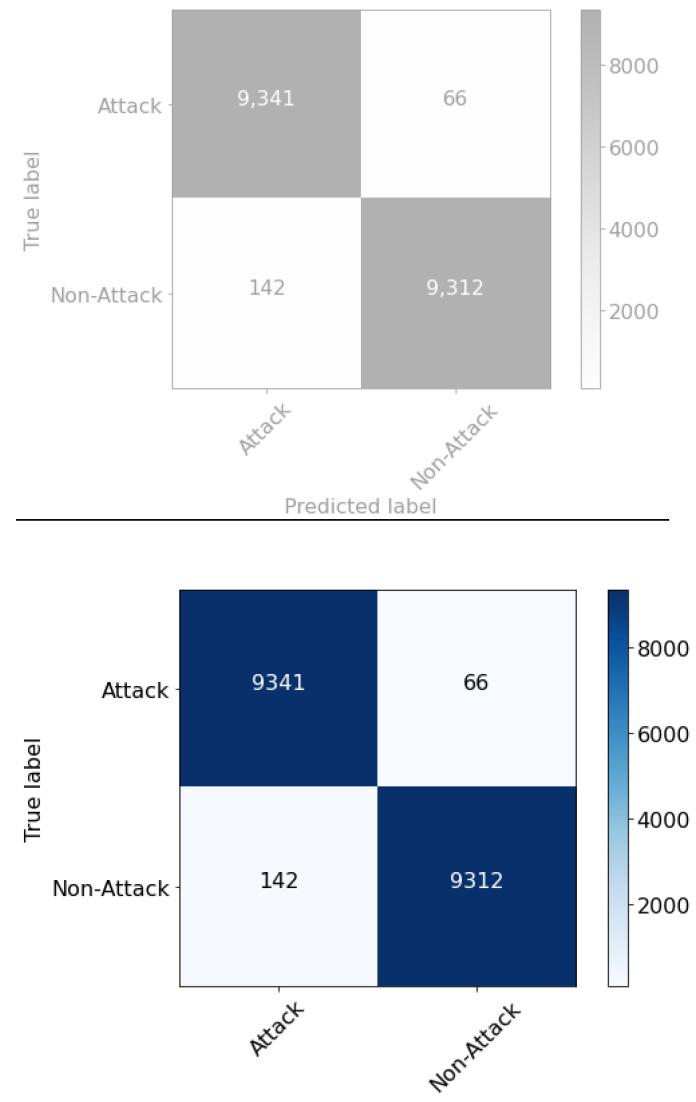
Confusion matrix for XGBoost.

After applying over-sampling and under-sampling techniques, there was a noticeable improvement in the results for all the classifiers in general. They achieved results that outperformed the benchmark study. However, the best performing classifiers were the XGBoost and LGBM. The difference between them is that when oversampled with the SMOTE technique, the XGBoost results were AUC: 0.9999, ACC: 0.9998, recall: 0.9996, precision: 1.0000, and F1: 0.9998. while the LGBM results were AUC: 1.0000, ACC: 0.9997, recall: 0.9994, precision: 1.0000, and F1: 0.9997. In contrast, the XGBoost results when undersampled with TomekLink were AUC: 1.0000, ACC: 0.9991, recall: 0.9912, precision: 0.9975, and F1: 0.9943. While LGBM results were AUC: 1.0000, ACC: 0.9993, recall: 0.9924, precision: 0.9987, and F1: 0.9956. In conclusion, after the experiment it was found that the XGBoost classifier combined with the oversampling technique achieved the best results (see Figure 2).

The XGBoost was applied with the default setting, except for one parameter, namely eval_metric = ‘mlogloss’. The training time complexity of XGBoost is O(KD ‖x‖ log(N)), where K is the number of trees generated, D represents the maximum depth of the tree, ‖x‖ represents the non-empty sample size for training and N represents the total number of records. Apparently, K and D, are constant numbers, and miss-values were filled in during the preprocessing step, so ‖x‖ would be equal to n in this case. So, the time complexity of XGBoost during training becomes O(N long(N)).

### 4.2. Explainable Artificial Intelligence (XAI)

**Figure 3:**
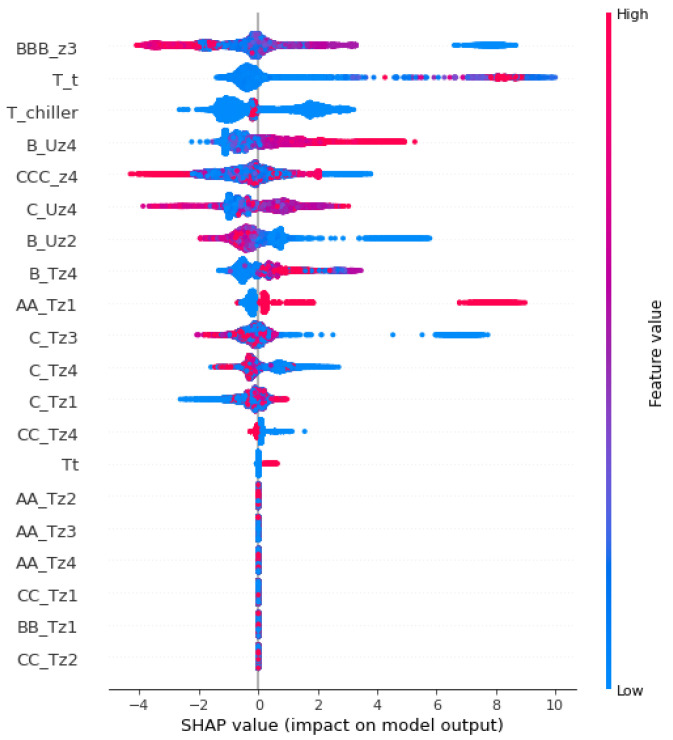
Global Explanation of XGBoost using SHAP.

**Figure 4:**
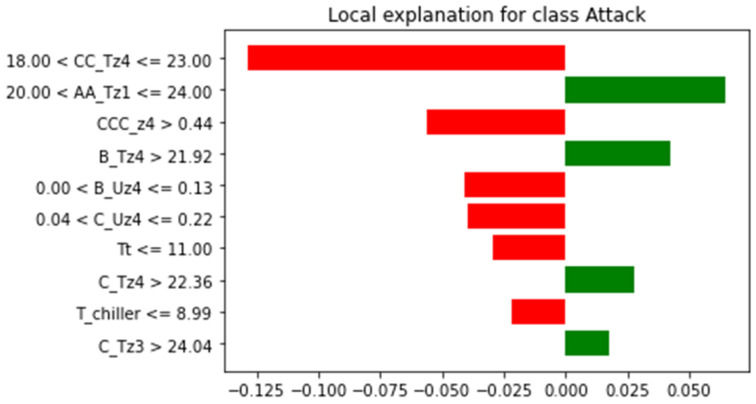
Local Explanation of XGBoost using LIME.

Experimental results indicate that XGBoost delivered the best results with the selected features after SMOTE oversampling. The nature of the XGBoost model is black box. Therefore, XAI was applied to make the high-performance model more transparent. In the ML model, some models like DT, Logistic Regression (LR), K-Nearest Neighbor (KNN) are simple and transparent models. While the complex models like SVM, ANN and ensemble models are opaque. In the current study post-hoc approach using LIME and SHAP was applied. Figure 3 provides the global explanation of the XGBoost model using selected features and an oversampled dataset. Figure 4 shows a local explanation of the XGBoost model using a selected and oversampled dataset. In addition, the rules are extracted from the XGBoost model using the global surrogate model. Extracted rules are mentioned below.

- IF (C_Uz4 > 0.218) && (AA_Tz1 <= 22.0) && (C_Uz4 <= 0.4) THEN response: 8309.0 | based on 19,759 samples
- IF (C_Uz4 <= 0.218) && (BBB_z3 > -0.058) && (C_Uz4 <= 0.145) THEN response: 12172.0 | based on 13,325 samples
- IF (C_Uz4 <= 0.218) && (BBB_z3 <= −0.058) && (B_Uz4 <= 0.18) THEN response: 7681.0 | based on 13,047 samples
- IF (C_Uz4 > 0.218) && (AA_Tz1 <= 22.0) && (C_Uz4 > 0.4) THEN response: 303.0 | based on 4387 samples
- IF (C_Uz4 > 0.218) && (AA_Tz1 > 22.0) && (BBB_z3 > −0.423) THEN response: 2.0 | based on 3721 samples
- IF (C_Uz4 <= 0.218) && (BBB_z3 <= −0.058) && (B_Uz4 > 0.18) THEN response: 5.0 | based on 2210 samples
- IF (C_Uz4 <= 0.218) && (BBB_z3 > −0.058) && (C_Uz4 > 0.145) THEN response: 2.0 | based on 132 samples
- IF (C_Uz4 > 0.218) && (AA_Tz1 > 22.0) && (BBB_z3 <= −0.423) THEN response: 1.0 | based on 2 samples

## 5. Further Discussion

The current research focuses on the attacks related to smart HVAC systems, which are considered to be a critical component of the building management system (BMS). We used HVAC system attack detection dataset published by Elnour et al. [8], which was acquired using the transient system simulation tool (TRNSYS). Furthermore, the performance of the proposed models was compared to the benchmark study, [8]. According to our findings, most of the classifiers, particularly the XGBoost classifier, outperformed the benchmark study across all the evaluation measures. Table 10 illustrates the comparison of the benchmark study with our proposed study.

The results show that the current study outperformed the benchmark study. Furthermore, in the Elnour et al. [8] study the performance of the model was evaluated using only two evaluation measures, namely precision and recall. However, in the current study several classification evaluation measures are used to ensure the performance of the proposed models and to provide a comprehensive evaluation. Elnour et al. [8] achieved the highest results with IF classification and PCA for feature selection with a recall of 0.6049 and a precision of 0.9001. In the current study the recall was 0.9996 and the precision was increased from 0.9001 to 1.000. In addition, the number of features was reduced using set forward feature selection techniques. The number of reduced features used in the proposed model is reduced to 50% compared to the benchmark study [8]. After performing the step forward feature selection technique, it was found that the highest result was obtained with the XGBoost classifier where AUC: 0.9891, ACC: 0.9835, recall: 0.8153, precision: 0.9753, and F1: 0.8882.

As previous shown in Table 1 the comparison of the related studies and in Table 10 the comparison of the proposed model and the benchmark study. Wu et al. [9] used the fusion-based technique for the hidden voice attacks. The study has achieved the success rate of 0.941. The significance of the study was that extensive experiments were performed. However, the experiments were conducted over-the-air. Therefore, the experiments need to be performed in more controlled environment. Moreover, in the study, 40,000 samples were used to train the model while, 1000 samples were used to test model performance. Furthermore, Munir et al. [10] proposed the attack detection for HVAC systems. The nature of the dataset is an imbalance and the study has achieved a recall is 0.80, which is significantly lower than the recall of the proposed model. However, the study by Novikova and Bestuzhev [11] has performed the unsupervised technique for the attack detection in HVAC system. The study also performed the visual evaluation using the ECG dataset. In addition, some studies have used NSL-KDD dataset, RF has outperformed with an accuracy of 0.9964 [15], while XGBoost has achieved the best results with an accuracy of 0.97. The NSL-KDD dataset contains the list of attacks on the network and is one of the widely used dataset for intrusion detection system. Additionally, UNSW-NB15 is another dataset for the intrusion detection. The dataset was used by [15,16], the highest accuracy of 0.99 was achieved with KNN. Despite this, the study achieved the significant results, but the KNN model is a lazy learner and require huge testing time. However, the detection time is one of the essential factors in the attack detection systems. In the proposed study an accuracy of 0.9998 was achieved, and the best performing algorithm is an eager learner.

Nevertheless, the result of the proposed study is significant, but it also suffers from some limitations. The current study examined the performance of the proposed models of single buildings. In order to generalize the proposed models, the datasets of multiple smart building systems need to be used to train and test the model.

## 6. Conclusions

Machine learning techniques have aided and eased the process of detecting and classifying attacks targeted toward the multi-zone HVAC system. In this paper, we trained several classifiers, namely Decision Tree, Random Forest, Gradient Boosting, Ada Boost, Light GBM, XGBoost, and CatBoost to detect different types of attacks. The features used to train the classifiers were selected using step forward feature selection. To overcome the problem of an imbalanced dataset, SMOTE and TomekLink for over and under-sampling was used to prevent potentially biased results. The highest results were obtained with the XGBoost classifier, which produced the best AUC, ACC, recall, precision, and F1 score of 0.9999, 0.9998, 0.9996, 1.000, and 0.9998, respectively. Therefore, the proposed study outperformed the previous benchmark study by achieving higher performance on various evaluation metrics while using fewer features. In the current study, XGBoost was the best performing model suffering from black-box nature, therefore, XAI was applied to make the model explainable. SHAP and LIME were used to generate the local and global explanation. Furthermore, the rules were extracted using a global surrogate model to represent the decision making in a form easily understood by human. In this paper, we only considered the attacks available in the HVAC dataset. However, there is a need to extend the work to include more types of attacks and to investigate the performance of the algorithms using other datasets. In future work, we plan to train classifiers to detect new attacks in order to develop more secure and robust systems.

## Figures and Tables

**Table 1:** Summary of the previous related studies.

Ref	Year	Technique	Dataset	No of Features	Results
[9]	2021	Fusion-based method	15 speech commands	136	Accuracy = 0.941
[10]	2017	Unsupervised anomaly detection	real data collected over 2.5 years period	-	Precision = 0.91 Recall = 0.80
[11]	2020	RadViz visualization algorithm	VAST Challenge 2016: Mini-Challenge dataset	-	-
[12]	2021	BMM, Correntropy	IoTBot-IDS	13	Accuracy = 0.992
[13]	2021	Functional Shape Analysis (FSA)	IoT (SWaT) system	-	-
[14]	2020	XGBoost	NSL-KDD	8	Accuracy = 0.97 Precision = 0.97 Recall = 0.968 F-Score = 0.968
[15]	2021	KNN	UNSW-NB15	9	Accuracy = 0.99 Precision = 0.9432 Recall = 0.998 F-score = 0.97
[16]	2021	Dynamic Feature Selector	NSL-KDD, UNSW-NB15	124	Accuracy = 0.9965 (NSL-KDD) Accuracy = 0.9276 (UNSW-NB15)
[17]	2021	Single Wavelet Entropy (WSE)	-	-	Accuracy = 0.965
[18]	2018	Wavelet transform and DNN	Synthetic simulated power system dynamics	-	Accuracy > 0.90
[19]	2021	CDBN	FDIA dataset	-	Accuracy = 0.984
[20]	2021	EMD-MFCC with multi kernel SVM	Three datasets 960, 1152, and 12624 sentences	12	Accuracy > 0.90
[8]	2020	IF	HVAC system Log1: 194301 Log2: 8840	Log1: 51 Log2: 65	Precision = 0.90 Recall = 0.61

**Table 2:** Log file details in the HVAC system dataset.

Log Title	Type	# of Features	# of Samples
Log 1	Normal	51	194,301
Log 2	Normal	65	32,161
Log 3	Normal and attack (16 different attacks with different severity measures)	65	8840

**Table 3:** Description of the common attributes category in the three log files.

No	Feature Name	Datatype
1	Hour of year	Numerical
2	Hour of day	Numerical
3	Sensor temperature measurements	Numerical
4	Control signals	Numerical
5	Temperature setpoints	Numerical
6	Comfort indices	Numerical
7	Total power usage	Numerical
8	Label (system status)	Boolean

**Table 4:** Selected features using step forward feature selection.

	Unit	Feature Name
Temperature Sensor Measurements	Floor-B	Tz2
		Tz4
	Floor-C	Tz1
		Tz3
		Tz4
	Chiller System	T_t
Control Signals	Floor-B	Uz4
	Floor-C	Uz4
Setpoints	Floor-A	Tz1
		Tz2
		Tz3
		Tz4
	Floor-B	Tz1
		Tz2
		Tz3
		Tz4
	Floor-C	Tz1
		Tz2
		Tz3
		Tz4
	Water Tank	Tt
	Chiller	T_chiller
Thermal Comfort Indices (PMV)	Floor-B	z3
	Floor-C	Z4

**Table 5:** Models’ parameter values after optimization.

Model	Parameter	Value
DT	max_depth	9
	random_state	42
Random Forest	random State	1
	n-estimators	100
	max-depth	15
	min_samples_split	5
Gradient Boosting	learning rate	0.05
	max_depth	3
	max_features	0.5
	random_state	42
Ada Boost	learning_rate	1.02
	n_estimators	13
Extreme Gradient	eval_metric	mlogloss
Light GBM	learning_rate	0.1
	max_depth	−1
Cat Boosting	iterations	5
	learning_rate	0.8

**Table 6:** Experiment I: Testing results of the proposed classifiers suing all the features in the original dataset.

Model	AUC	ACC	Recall	Precision	F1
DT	0.9880	0.9962	0.9781	0.9746	0.9763
RF	0.9990	0.9923	0.9052	0.9987	0.9496
GB	0.9583	0.9822	0.7813	0.9969	0.8760
AB	0.9986	0.9915	0.9137	0.9792	0.9453
XGBoost	0.9992	0.9991	0.9915	0.9976	0.9945
LGBM	0.9990	0.9995	0.9964	0.9976	0.9970
CB	0.9957	0.9914	0.9016	0.9907	0.9440

**Table 7:** Experiment II: Testing results of the proposed classifiers after applying step forward feature selection.

Model	Feature Selection	No. of Features	AUC	ACC	Recall	Precision	F1
DT	Step Forward feature selection	24	0.9045	0.9733	0.8226	0.8410	0.8317
RF			0.9714	0.9814	0.7679	1.0000	0.8687
GB			0.9429	0.9673	0.5966	0.9939	0.7456
AB			0.9601	0.9690	0.6440	0.9550	0.7692
XGBoost			0.9891	0.9835	0.8153	0.9753	0.8882
LGBM			0.9865	0.9830	0.7983	0.9880	0.8831
CB			0.9426	0.9769	0.7339	0.9711	0.8360

**Table 8:** Experiment III: Testing results of the classifiers after SMOTE algorithm using selected features.

Classifier:	AUC	ACC	Recall	Precision	F1
DT	0.9980	0.9980	0.9979	0.9982	0.9980
RF	1.0000	0.9994	0.9992	0.9997	0.9994
GB	0.9976	0.9769	0.9628	0.9909	0.9766
AB	0.9986	0.9835	0.9779	0.9891	0.9835
XGBoost	0.9999	0.9998	0.9996	1.0000	0.9998
LGBM	1.0000	0.9997	0.9994	1.0000	0.9997
CB	0.9986	0.9864	0.9855	0.9874	0.9864

**Table 9:** Experiment IV: Testing results of the classifiers after TomekLinks algorithm using selected features.

Model	AUC	ACC	Recall	Precision	F1
DT	0.9949	0.9981	0.9912	0.9849	0.9880
RF	0.9994	0.9928	0.9104	0.9986	0.9524
GB	0.9606	0.9830	0.7891	0.9952	0.8803
AB	0.9978	0.9912	0.8965	0.9916	0.9416
XGBoost	1.0000	0.9991	0.9912	0.9975	0.9943
LGBM	1.0000	0.9993	0.9924	0.9987	0.9956
CB	0.9963	0.9928	0.9306	0.9775	0.9534

**Table 10:** Comparison of the proposed work with the benchmark study.

Study	Data Partitioning	No. of Features	Model	AUC	ACC	Recall	Precision	F1
[8]	5-Fold cross Validation	50	IF	-	-	0.6049	0.9001	-
Our Study	Hold out (75–25)	24	XGBoost	0.9999	0.9998	0.9996	1.0000	0.9998

## Data Availability

The study used an open-source dataset.
